# Measuring spatiotemporal accessibility and equity of emergency medical services in Shanghai, China

**DOI:** 10.1371/journal.pone.0322656

**Published:** 2025-05-08

**Authors:** Haolin Zhu, Mo Xu, Luying Zhu

**Affiliations:** 1 School of Sciences for Human Habitat, University of Chinese Academy of Sciences, Beijing, China; 2 Beijing Emergency Medical Center, Beijing, China; Huazhong University of Science and Technology, CHINA

## Abstract

Emergency Medical Services (EMS) are a critical component of public healthcare. This study aimed to precisely measure the spatiotemporal accessibility and equity of EMS in Shanghai. We constructed a research model that combines web mapping application programming interfaces (APIs) and empirical Bayesian kriging (EBK) interpolation methods to calculate EMS accessibility, and used the Theil index to assess the equity of EMS accessibility. Additionally, we used the Minimum Cumulative Resistance (MCR) Model to evaluate the rationality of EMS stations layout. The results indicated that the per capital response time (PRT) in Shanghai increased from 11.46 minutes during smooth traffic periods to 13.08 minutes during peak traffic periods, and the 12 min EAI population coverage rate dropped from 60.6% to 41.9%. Thile index results demonstrated that there is significant inequity in Shanghai’s EMS, primarily driven by the disparity in accessibility between central and suburban districts. The results based on the MCR model indicated that the EMS stations in central districts had smaller shortest-time service areas, fewer resource demands, and lower PRTs. Obtained results revealed that EMS accessibility in Shanghai exhibited clear spatiotemporal variation, driven by disparities across urban districts and multi-period traffic conditions. Additionally, the irrational layout of EMS stations exacerbated the equity and opportunity to EMS services for citizens in suburban districts of Shanghai.

## Introduction

Emergency Medical Services (EMS) are a critical component of public healthcare [1,2] that provide immediate medical care in emergencies, thereby significantly reducing morbidity and mortality rates [2–4], particularly in cases of accidents, sudden illnesses, and major disasters. From the perspective of social equity, each person should have equal time, costs, and opportunities to access such services [5,6]. However, in reality, EMS face numerous challenges, such as long distances, poor traffic conditions, and insufficient emergency resources [7–9]. These obstacles can lead to preventable deaths and disabilities and exacerbate health disparities and social inequities [10,11].

Quantifying spatial accessibility is the foundation for measuring spatial equity in provision and acquisition of EMS [12,13]. As such, the spatial inequity and urban stratification heterogeneity of public services based on accessibility has been widely studied [13–17]. In urban studies, the concept of accessibility is primarily based on Hansen’s theory, which defines accessibility as the opportunity that an individual or type of person at a given location possesses to take part in a particular activity or set of activities [18]. This definition emphasizes the spatial attributes of accessibility, including origin, destination (opportunity), and spatial impedance [18].

Studies on accessibility have predominantly applied four main categories: opportunity-, gravity-, person-, and utility-based methods [12,19,20]. Among them, the GIS-based network analysis [21–23] and the two-step floating catchment area model [24–26] has been widely adopted in the research on accessibility. Although a GIS-based network analysis is precise for calculating road lengths, it often neglects real-time traffic conditions [12,27]. The two-step floating catchment area model uses travel time functions instead of distance coefficients to calculate accessibility. Although it is easy to implement and is highly scalable, it is still limited in effectively calculating accurate EMS response times and capturing real-time traffic impacts [28]. At the same time, the calculation of the spatiotemporal distribution of EMS accessibility is based on the identification of urban spatial stratification heterogeneity [17]. To address these limitations, this study utilized the application programming interface (API) of a navigation platform as the data sampling source. The API, provided by map service platforms, offers an interface that allows users to programmatically access geographic data services [29,30]. These API data are highly accurate and updated in real-time, providing a solid foundation for the quantitative calculation of urban spatiotemporal accessibility [31].

This study aims to analyze EMS station layouts using the cost-allocation method. Traditional methods, such as the Tessellation polygon method [32], which use Euclidean distance as the cost metric, are limited in accounting for the influence of urban spatial heterogeneity [33]. The Minimum Cumulative Resistance (MCR) Model, noted for its effectiveness in identifying urban spatial resistance, offers greater precision [34,35]. It has been widely applied in research of urban land-use zoning and urban minimum cumulative resistance boundaries [36]. The cost-allocation results based on the MCR model are crucial for our study on resource allocation and the efficiency of all EMS stations.

Therefore, this study aimed to conduct precise research on EMS accessibility by integrating web mapping APIs and empirical Bayesian kriging (EBK) interpolation. Additionally, we adopted a multiscale research perspective encompassing “citywide-district-station,” with several quantitative accessibility indicators to evaluate spatiotemporal variations in EMS accessibility, the equity of the distribution of EMS accessibility across each district, and the rationality in the layout of EMS stations.

## Methods

### Study area

**Fig 1:**
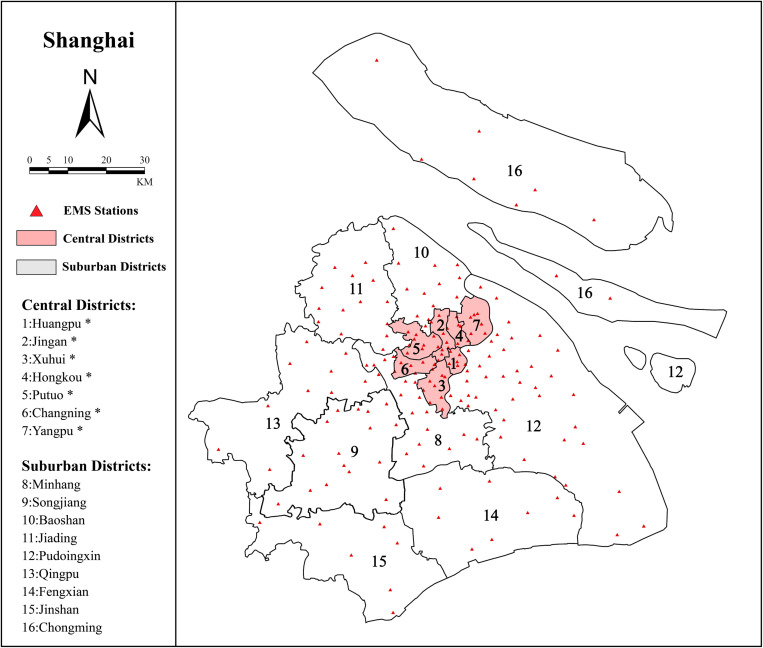
The information of districts and EMS stations across Shanghai.

This study focused on Shanghai, a city with a leading prehospital EMS system in China [37]. Shanghai consists of 16 administrative districts, seven of which are located in the central urban area and cover only 4.6% of the total area of Shanghai [38](Fig 1). According to data released by the Shanghai Emergency Medical Center, the per capita response time in the central urban districts of Shanghai was reduced to 9 min and 28 s. The current EMS target is to achieve a citywide per capita response time (PRT) of less than 12 min and allocate one ambulance team per 30,000 people. However, most EMS stations in Shanghai are located in existing hospitals and community health centers. This has led to an uneven allocation of EMS stations across different areas of the city.

The EMS process can be divided into the following phases [39]: (1) dispatch time, which is the time taken from receiving the emergency call to the departure of an ambulance; (2) driving time, which is the time taken from the ambulance’s departure to its arrival at the scene; (3) transport time, which is the time taken from on-site treatment to transportation to the hospital; and (4) return time, which is the time taken for the ambulance to return to the emergency station from the hospital. The response time includes both the “dispatch time” and the “driving time.” [40] According to the Guiding Opinions on Further Improving Prehospital EMS issued by the National Health Commission of the People’s Republic of China on September 4, 2020 (http://www.nhc.gov.cn/yzygj/s3594q/202009/4b20d1ac72914b3997f76110ccc0103d.shtml), by 2025, 95% of EMS dispatch times should not exceed 3 min. We believe that as a leading city in terms of economic development and urban growth, Shanghai’s average EMS preparation time should be shorter than the national upper limit for EMS preparation. For the sake of calculation, we set the average EMS preparation time in Shanghai to 2 min. Therefore, our calculated results included EMS driving time plus an additional 2 min.

### Data sources

The administrative boundary map of Shanghai was created by us using ArcGIS Pro 3.0, with information sourced from the standard map data released by the Shanghai Planning and Natural Resources Bureau (https://hd.ghzyj.sh.gov.cn/ch/bzd).A total of 188 EMS stations in Shanghai were obtained from the Shanghai Medical Emergency Center (https://www.sh120.sh.cn/shsjjzx/jjzdfbt/jjzdfbt.html) on May 28, 2024. The coordinates of these EMS stations were collected using Amap Coordinate Picker (https://lbs.amap.com/tools/picker).

This study’s analysis of EMS supply-demand matching relied on high-quality population distribution data. The LandScan HD dataset and the WorldPop Constrained 2020 dataset are the most commonly used population dataset, both offering high spatial resolution. However, LandScan HD, with its higher update frequency and 24-hour comprehensive population distribution calculations, provides advantages for multi-period analysis, especially in non-residential areas such as public, office, and transportation spaces. As a result, population distribution data for Shanghai were sourced from the “LandScan HD China v1.0 2022” dataset [41]. The coordinates of the EMS destinations were derived from LandScan population distribution data and plotted using resampling and raster-to-point tools in ArcGIS Pro 3.0. The EMS travel times for Shanghai EMS stations were obtained using the Amap API route-planning function, capturing real-time traffic data on May 28, 2024, at 00:00, 12:00, 16:00, and 18:00 on a working day.

### Methodology framework

**Fig 2:**
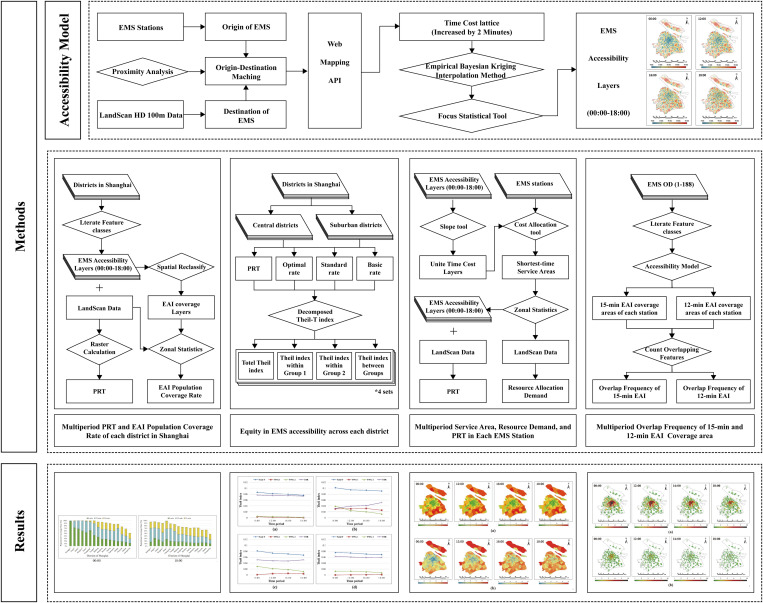
Research Framework.

We established a multilevel methodology framework to analyze the spatiotemporal accessibility and equity of EMS in Shanghai and developed an EMS layout evaluation model, aiming to provide a comprehensive assessment of Shanghai’s EMS system. The methodology framework is divided into three main components: the EMS accessibility calculation method, the spatial equity calculation method, and the EMS layout analysis method (Fig 2).

(1) EMS Accessibility calculation method: This study developed an accessibility model by integrating web mapping APIs with the EBK interpolation method, providing high-precision spatiotemporal distribution results for EMS accessibility in Shanghai. Additionally, by combining accessibility results with population distribution data, we innovatively proposed methods for calculating two accessibility indicators: per capita EMS response time (PRT) and EMS accessibility isochrone (EAI) population coverage rate. The former is essential for evaluating the citywide average EMS efficiency, while the latter is crucial for assessing EMS coverage quality. (2) Spatial Equity calculation method: Based on the accessibility indicators, this study employed a population-weighted Theil index to quantify spatial equity disparities in EMS accessibility across all districts in Shanghai and traced the sources of inequity. (3) EMS stations layout analysis method: Using the MCR model, we calculated the shortest-time service range for each EMS station in Shanghai. We then integrated population data and accessibility results to assess resource demands and PRT for each station. Furthermore, we evaluated the planning distance between EMS stations using the EAI overlap model to assess the rationality of Shanghai’s EMS stations layout.

### Calculating accessibility through web mapping APIs

This study used the LandScan dataset to identify the spatial distribution of urban populations. The space was divided into spatial cells with a resolution of 250 m x 250 m, with their center points considered as EMS destinations. All EMS stations in Shanghai were considered EMS origins.

Determining the shortest EMS driving time for each destination requires matching the destination with multiple nearby EMS stations and utilizing web mapping API for sampling and comparison. However, increasing the number of matching groups leads to higher data access volumes, which, in turn, reduces sampling efficiency and affects the accuracy of the results. Therefore, based on the group settings referenced in related literature [42], the number of origin-destination (OD) matching groups was set to three. We used the Generate-Near-Table tool in ArcGIS Pro 3.0 to match each EMS destination to its three nearest EMS stations to generate the OD coordinate information. The EMS driving times from stations to destinations were obtained using Amap APIs at four distinct time periods on weekdays: smooth traffic period (00:00), regular traffic period (12:00), secondary peak traffic period (16:00), and peak traffic period (18:00). The shortest EMS driving time for all destinations was recorded, and an additional 2 min was added to account for the EMS dispatch time, resulting in the final EMS response time dot matrix data. Using this data, we employed an EBK interpolation method to create a raster map of EMS response times in Shanghai. Finally, we used a focal statistics tool to remove outliers and applied the LandScan data as a mask to generate the final multiperiod EMS accessibility layers for Shanghai, with a resolution of 100 m x 100 m.

### Calculating the PRT and population coverage rate of EAI

In urban EMS, two indicators receive the most public attention and are widely used to measure the quality and efficiency of these services: the per capita emergency response time and the population coverage rate within a specified emergency response time. The former represents the average quality of emergency services in an area, while the latter indicates the proportion of the population receiving different levels of service quality. This chapter combined accessibility layers and population distribution data to develop an PRT calculation method and used an isochrone model to calculate the EAI population coverage rate. The PRT $\overset{―}{R}$ for Shanghai can be obtained. The formula is as follows:


$$\overset{―}{R}=\frac{\sum_{i=1}^{n}P_{i}*R_{i}}{\sum_{i=1}^{n}P_{i}}$$

where *n* is the number of spatial units in the study area, $P_{i}$ is the population of spatial unit *i*, and $R_{i}$ is the response time of spatial unit *i*.

We converted the accessibility layer into vector polygons at 1 min intervals to generate EAIs. We then calculated the proportion of the total population covered by the different EAIs in Shanghai, resulting in the population coverage rate for each EAI.

### Calculating inequity of the distribution of EMS accessibility across each district using Theil index

In this study, we used the Theil index to assess the equity of EMS accessibility across different districts. This method could effectively measure overall inequality and breaks it down into within-group and between-group inequalities, making the analysis more accurate and representative [43]. In this chapter, we categorized all districts in Shanghai into two groups: central districts (Group 1) and suburban districts (Group 2). We used the PRT and 9 min, 12 min, 15 min EAI population coverage rate of each district as our indicators, combined with the population of each district, to calculate the overall Theil index T for all districts in Shanghai, the within-group Theil index $T_{WG}1$ for group 1, and the within-group Theil index $T_{WG}2$ for group 2, and the between-group Theil index $T_{BG}$, the calculation formula was as follows:


$$Y_{ij}=P_{ij}*\overset{―}{R_{ij}}$$

$$Y_{i}=\sum_{j}Y_{ij}\;$$

where $Y_{ij}$ is the total response time of all citizens (or the population covered by EAIs, similarly hereafter) of district *j* in group *i*, $Y_{i}$ is the total response time of all citizens in group *i,* $P_{ij}$ is the population of spatial unit *k* of district *j*, and $R_{ij}$ is the per capital response time (or the EAIs population coverage rate) of district *j* in group *i*.


$$T=\sum_{i}\sum_{j}\left((\frac{Y_{ij}}{Y})\ln(\frac{Y_{ij}/Y}{P_{ij}/P})\right)$$

$$T_{i}=\sum_{j}\left((\frac{Y_{ij}}{Y_{i}})\ln(\frac{Y_{ij}/Y_{i}}{P_{ij}/P_{i}})\right)$$

where *T* is total Theil index, $T_{i}$ is Theil index within group *i*, *Y* is the total response time of all citizens (or all the population coverage within EAIs) in the city, $P_{ij}$ is the population of district *j* in group *i*, and $P_{i}$ is the population of group *i*, *P* is the population in the city.


$$T=\sum_{i}(\frac{Y_{i}}{Y})T_{i}+\sum_{i}\left((\frac{Y_{i}}{Y})\ln\left(\frac{\frac{Y_{i}}{Y}}{\frac{P_{i}}{P}}\right)\right)=T_{WG}+T_{BG}$$

where $T_{WG}$ is the Theil index within the groups, $T_{BG}$ is the Theil index between the groups.

### Calculating shortest-time services range, resource demand, and PRT of each EMS station

This chapter aimed to calculate the shortest-time service range of each EMS station using the MCR model and to evaluate current resource demands and PRT by integrating population distribution data with accessibility layers. These results could assess the equity of resource allocation and service quality of each station, thereby evaluating the rationality of the current EMS layout.

The MCR model can identify resistance values for urban spatial units and calculate the multi-period minimum cumulative resistance boundaries for all EMS stations. In this study, urban spatial resistance is defined as the time cost required for EMS vehicles to traverse each spatial unit (100m x 100m). First, using the accessibility layers previously calculated, we applied the Surface-Parameters tool in ArcGIS PRO 3.0 to calculate the spatial impedance layers (s/100m). Second, based on this cost layer, we used the cost allocation tool in ArcGIS PRO 3.0 to determine the shortest-time service area for each EMS station. This cumulative time-cost-based partitioning method is more precise than traditional methods based on the Euclidean distance (such as Thiessen polygons) and more effectively considers the impact of different road networks and traffic conditions.

To calculate the EMS resource demand $D_{i}$ for each station *i*, the calculation formula was as follows:


$$D_{i}=P_{i}*Z$$

where $P_{i}$ is the population served by EMS station *i*, and Z is the per capita EMS resource demand (one ambulance team per 30,000 people).

To calculate the PRT $\overset{―}{R_{j}}$ of each station *j*, the calculation formula was as follows:


$$\overset{―}{R_{j}}\;=\frac{\sum_{i=1}^{n}P_{ij}*R_{ij}}{\sum_{i=1}^{n}P_{ij}}$$

where $P_{ij}$ is the population of spatial cell *i* in the service area of station *j*, $R_{ij}$ is the response time of spatial cell *i* in the service area of station *j*.

### Calculating EAI overlap frequency of all EMS stations

In this section, we considered each EMS station as the origin and matched them with all EMS destinations within an appropriate range to calculate the 12–15-min EAI coverage area for each station. Initially, we set the OD sampling radius to 5 kilometers. However, after calculations, we found that the 15 min EAI coverage area during smooth traffic periods in Shanghai exceeded this range. As a result, the OD sampling radius was increased to 8 kilometers. The calculation results indicate that this range can fully encompass the 12–15-min EAI coverage area of all EMS stations.

Thus, we used all EMS stations in Shanghai as EMS origins and matched all EMS destinations within an 8 km radius of each station. On June 7, 2024, we recalculated the travel times using Amap API’s route planning function and used the previously mentioned calculation method to construct a multiperiod 12 min and 15 min EAI for each EMS station. We then counted the overlap frequency in their coverage areas to identify regions in which the spacing between EMS stations was irrational.

## Results

### Multiperiod EMS accessibility layers in Shanghai

**Fig 3:**
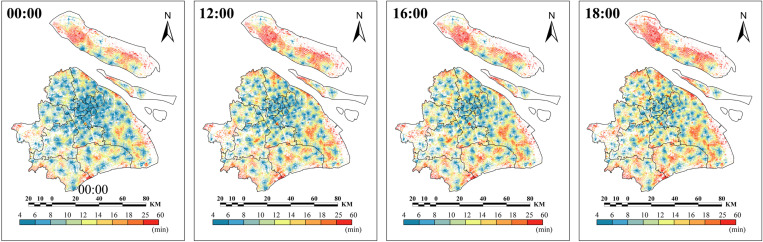
Multiperiod EMS accessibility in Shanghai.

The results showed that EMS accessibility in Shanghai exhibited significant spatiotemporal variation (Fig 3). At 00:00, the EMS response time in most areas of the central urban district was less than 12 min. However, suburban areas had noticeably poorer EMS accessibility than that of central districts. The results also showed that the northern and western parts of Chongming had the lowest EMS accessibility. At 18:00, there was a noticeable decline in EMS accessibility across the entire city, with the central districts experiencing a more pronounced decrease than that of the suburban areas.

### Multiperiod PRT and EAI population coverage rate in Shanghai

The calculation results showed that during the smooth traffic period at 00:00, the PRT in Shanghai was 11.46 min, increased to 12.23 min by 12:00, further rose to 12.75 min by 16:00, and reached its peak at 18:00, rising to 13.08 min.

**Fig 4:**
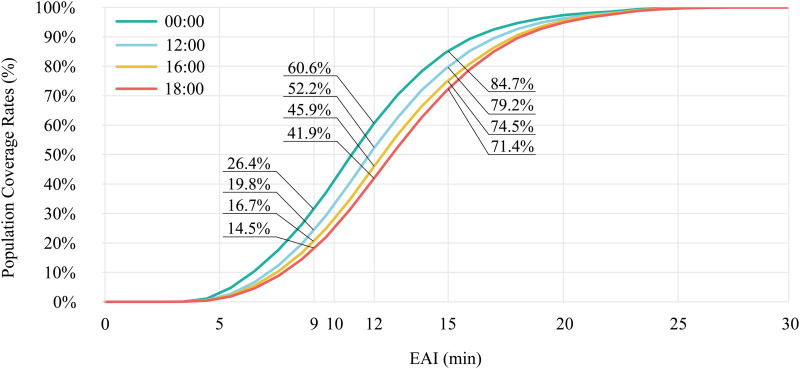
Multiperiod EAI population coverage rate in Shanghai.

The calculation results (Fig 4) showed that the 9 min, 12 min, and 15 min EAI population coverage rates decreased from 26.4%, 60.6%, and 84.7% at 00:00 to 19.8%, 52.2%, and 79.2% at 12:00; 16.7%, 45.9%, and 74.5% at 16:00; and 14.5%, 41.9%, and 71.4% at 18:00, respectively.

### Multiperiod PRT and EAI population coverage rates of each district across Shanghai

The results showed a significant discrepancy in PRTs across various districts in Shanghai (Table 1). At 00:00, Huangpu had the lowest PRT (7.15 min), followed by Jing’an and Xuhui (8.47 min and 8.55 min). Among the central districts, Yangpu had the highest PRT (9.86 min), which was considerably lower than that of the suburban district with the lowest PRT, Minhang (10.65 min). Chongming had the highest PRT (16.32 min).

**Table 1:** Multiperiod PRT of each district across Shanghai.

District	00:00 (min)	12:00 (min)	16:00 (min)	18:00(min)
**Huangpu** ^*^	7.15	8.99	9.51	11.49
**Jingan** ^*^	8.47	9.39	10.01	10.95
**Xuhui** ^*^	8.55	9.96	10.64	11.36
**Hongkou** ^*^	8.92	10.37	10.88	12.12
**Putuo** ^*^	9.14	9.88	10.35	11.13
**Changning** ^*^	9.29	10.31	11.00	11.71
**Yangpu** ^*^	9.86	10.99	11.56	12.10
**Minhang**	10.65	11.64	12.24	12.90
**Songjiang**	11.22	12.00	12.72	12.94
**Baoshan**	11.35	12.13	12.64	13.16
**Jiading**	11.75	12.43	12.95	13.60
**Pudongxin**	12.02	12.74	13.45	13.72
**Qingpu**	12.28	13.12	13.51	13.66
**Fengxian**	12.85	13.63	14.06	13.95
**Jinshan**	13.74	14.10	14.30	14.28
**Chongming**	15.67	16.22	16.23	16.32

The districts are arranged in ascending order based on their PRT at 00:00. The

*represents the central districts. PRT, per capital response time.

At 18:00, the PRTs across all districts in Shanghai increased owing to traffic conditions. Huangpu experienced the highest increase (60.8%), followed by Hongkou (35.9% increase) and Xuhui (32.8% increase), whereas Chongming saw a minimal increase of only 4.2%. Jing’an (10.95 min) became the district with the lowest PRT in Shanghai, followed by Putuo and Xuhui (11.13 min and 11.35 min). Although the central districts were more significantly affected by traffic, the highest PRT in the central districts was in Hongkou (12.12 min), which still remained notably lower than that of the lowest suburban district, Minhang (12.90 min).

There was also a significant disparity in the population coverage rates of EAI among the urban districts during the different periods.

**Fig 5:**
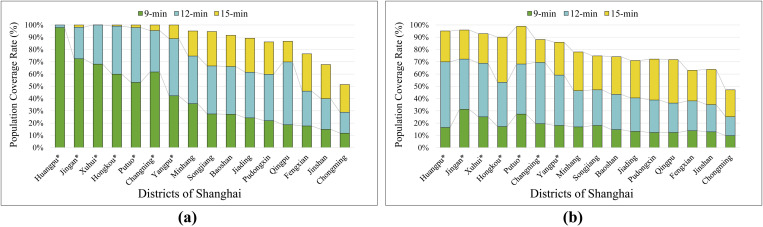
The 9 min, 12 min, 15 min EAI population coverage rates across each district in Shanghai. (a) EAIs population coverage rate during 00:00. (b) EAIs population coverage rate during 18:00.

(1) At 00:00 (Fig 5a), Huangpu led the city with a 9 min EAI population coverage rate of 97.8%, followed by Jing’an and Xuhui (72.4% and 68.1%, respectively). Among the central districts, Yangpu had the lowest coverage rate (42.2%), which was still significantly higher than that of the highest suburban district, Minhang (35.8%). The districts with the lowest coverage rates were Jinshan (14.9%) and Chongming (11.8%).
(2) The 12 min EAI population coverage rate was highest in Huangpu, reaching 100%. The lowest coverage rate among the central districts was in Yangpu (88.9%), which was higher than that of the highest suburban district, Minhang (74.6%). The districts with the lowest coverage rates were Jinshan and Chongming (40.0% and 28.9%, respectively).
(3) The lowest 15 min EAI population coverage rate among the central districts was in Yangpu (99.8%), whereas the other six central districts achieved 100% coverage. Among the suburban districts, Minhang had the highest 15 min EAI population coverage at 95.1%. The districts with the lowest coverage rates were Jinshan and Chongming (67.7% and 51.4%, respectively).

By 18:00 (Fig 5b), the population coverage rates of EAIs in all districts decreased:

(1) The highest 9 min EAI population coverage rates were in Jing’an and Putuo (31.3% and 27.4%, respectively), followed by Xuhui and Yangpu (25.1% and 18.1%, respectively). The lowest rates were observed in Pudongxin and Chongming (12.4% and 9.8%, respectively).
(2) For the 12 min EAI, the highest population coverage rates were in Jing’an and Huangpu (72.2% and 69.9%, respectively), followed by Changning and Xuhui (69.5% and 68.8%, respectively). The lowest rates were observed in Jinshan and Chongming (35.1% and 25.2%, respectively).
(3) Within the 15 min EAI, the highest population coverage rates were in Putuo and Jing’an (98.9% and 95.9%, respectively), followed by Huangpu and Xuhui (95.1% and 92.8%, respectively). The lowest rates were observed in Fengxian and Chongming (63.1% and 47.2%, respectively).

### Theil indexes of four sets EMS accessibility indicators across each district

To calculate the inequity of EMS accessibility across all districts, this chapter used PRT, 9 min, 12 min, and 15 min EAI population coverage rates as indicators for the Theil index. The PRT could represented per capital accessibility, and the 9 min, 12 min, and 15 min EAI population coverage rates could represent the proportions of the population that could receive EMS of optimal, standard, and basic quality levels, respectively. The Theil index for EMS accessibility was calculated by weighting the population of each group and district.

**Fig 6:**
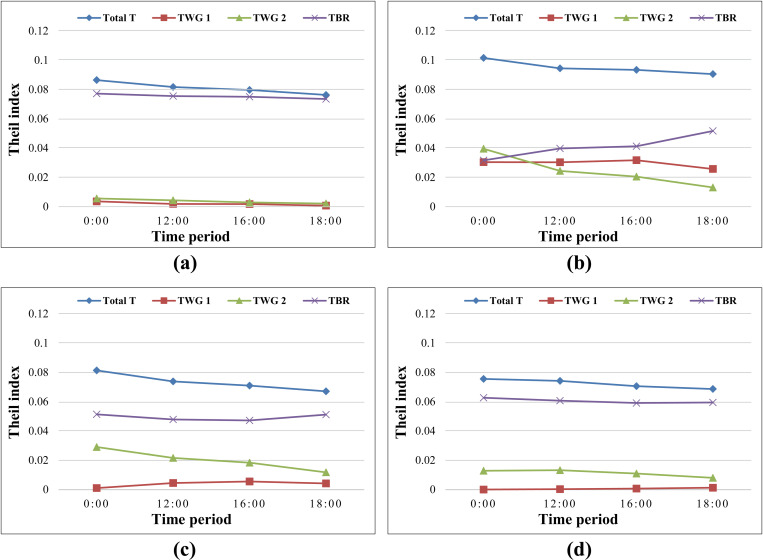
The Theil indexes for four sets of EMS accessibility indicators. (a) The Theil indexes for PRT. (b) The Theil indexes for EMS optimal rate. (c) The Theil indexes for EMS standard rate. (d) The Theil indexes for EMS basic rate.

As shown in Fig 6, by 00:00, the four sets of TTIs (PRT, EMS optimal rate, EMS standard rate, and EMS basic rate) were 0.086, 0.101, 0.081, and 0.076, respectively. This indicated significant inequity in EMS accessibility across districts, with the highest inequity in the EMS optimal rate. Furthermore, all four TTIs dropped (to 0.076, 0.091, 0.067, and 0.068, respectively) by 18:00 due to traffic congestion, with the TTI of the EMS standard rate showing the highest reduction rate.

In the four sets of Theil indexes, the contribution rates of TWG and TBG varied. The calculation results of the Theil index for PRT showed that by 00:00, TWG1, TWG2, and TBG were 0.003, 0.005, and 0.077, respectively. This indicates that the inequity of PRT was mainly between groups. By 18:00, TWG1, TWG2, and TBG had decreased to 0.001, 0.002, and 0.074, respectively. This represents a reduction in overall PRT inequity. However, the between-group disparity remained significant.

The Theil index of the EMS optimal rate showed that by 00:00, TWG1, TWG2, and TBG had similar values (0.030, 0.040, and 0.032, respectively), indicating that the inequity in the EMS optimal rate was present both within and between groups. By 18:00, TWG1 and TWG2 dropped to 0.026 and 0.013, respectively, while TBG increased to 0.051. This indicated that the between-group inequity increased, and the inequity within the group1 became greater than that within the group2.

The Theil index of the EMS standard rate showed that by 00:00, TWG1, TWG2, and TBG were 0.001, 0.029, and 0.051, respectively. This indicated that the inequity in the EMS standard rate was mainly between groups and within Group 2. By 18:00, TBG remained almost unchanged at 0.051, while TWG1 and TWG2 decreased to 0.013 and 0.026, respectively. This suggested that, the between-group inequity remained largely unchanged, while the inequity within Group 1 became greater than that within Group 2.

The Theil index of the EMS basic rate showed that by 00:00, TWG1, TWG2, and TBG were 0, 0.013, and 0.063, respectively. This indicates that the inequity in the EMS basic rate was primarily between groups, followed by within Group 2, while Group 1 was completely fair internally. By 18:00, TWG1 rose to 0.001, and TWG2 and TBG dropped to 0.008 and 0.059, respectively. This suggested that, the inequity appeared within the central districts, while the between-group disparity remained the main source of inequity.

### Multiperiod shortest-time service range, resource demand, and PRT of each EMS station.

**Fig 7:**
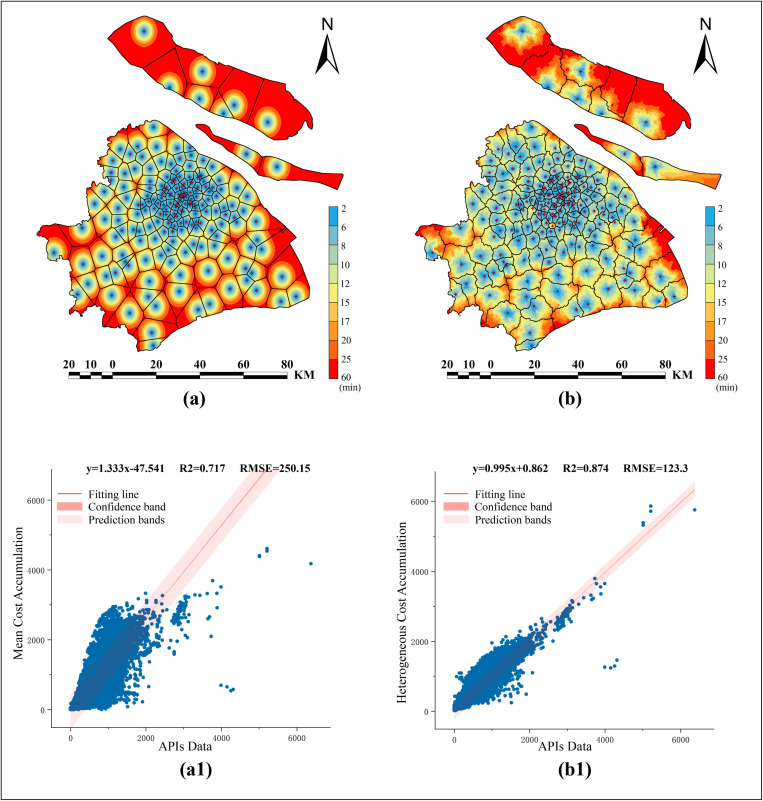
Partitioning results based on two cost-allocation methods. (a) Accumulated results and the corresponding tessellation polygon boundary based on the mean spatial cost. (a1) Fitting performance of the Mean Cost Accumulation Model. (b) Cost accumulation results and MCR-derived boundaries based on heterogeneous spatial costs. (b1) Fitting performance of the Heterogeneous Cost Accumulation Model.

Since the Tessellation polygon method assumes urban space to be a homogeneous environment for partitioning, whereas our MCR model incorporates heterogeneous spatial costs, we employed both methods for distance cost accumulation to calculate the least-cost cumulative boundaries. The accumulated cost and the corresponding Tessellation polygon boundary based on the mean cost were illustrated in Fig 7a, while the cost accumulation results and the MCR-derived boundaries incorporating heterogeneous spatial costs were shown in Fig 7b. We considered the APIs data as the ground truth and conducted a comparative analysis of the accumulated results from both models using linear regression. The results indicated that, compared to the mean cost accumulation method (R² = 0.717, RMSE = 250.2) (Fig 7a1), the MCR model incorporating heterogeneous spatial costs demonstrates superior accuracy (R² = 0.874, RMSE = 123.3) (Fig 7b1). This highlighted the significant advantage of the MCR model with heterogeneous cost considerations over the Tessellation polygon method in shortest-time service range for each EMS station.

**Fig 8:**
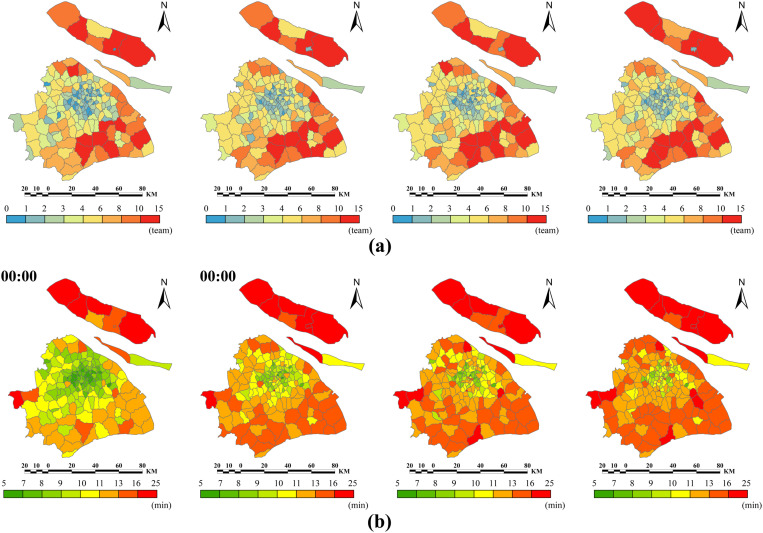
Multiperiod shortest-time service range, resource demand, and PRT of each EMS station. (a) The shortest-time service range and resource demand of each EMS station. (b) The PRT of each EMS station.

The results indicate that the average EMS resource demand for all EMS stations across Shanghai is 4.4 ambulance teams; however, Tairi station in Fengxian had the highest emergency resource demand, reaching 14.7 teams. Additionally, 16 stations (8.5%) located in eastern and southern Fengxian, eastern Pudongxin, northern Chongming, eastern Songjiang, northern and southern Jinshan, central Qingpu, and northern Baoshan required more than ten teams (Fig 8a).

**Fig 9:**
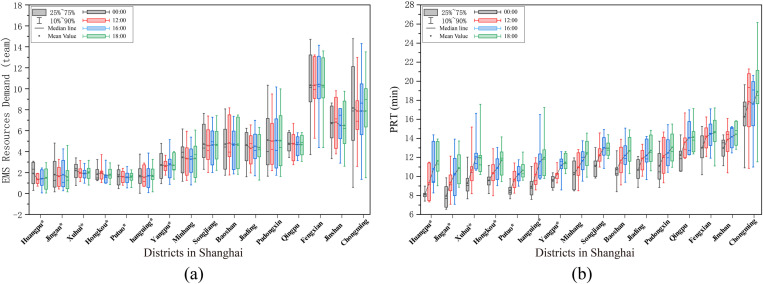
The EMS resource demand and PRT of each EMS station. (a) The EMS resource demand. (b) The PRT of each EMS station.

The statistical results showed that the average resource demand per EMS station in central districts was 2.0 teams, whereas that in suburban districts was 5.3 teams. Jing’an had the lowest demand (1.7 teams), and Fengxian had the highest demand (10.5 teams) (Fig 9a). Additionally, only a few EMS stations experienced changes in their shortest-time service areas owing to periodic traffic, which consequently affected their resource demands.

The results indicated that PRTs of EMS stations in suburban districts were significantly higher than those in central districts, with Chongming being particularly elevated (Fig 8b). At 00:00, the average PRT of all EMS stations citywide was 11.1 min, with central districts averaging 8.8 min and suburban districts averaging 11.5 min. Nine EMS stations (5.9%) in northern Chongming, eastern Pudong, western Qingpu, and western and southern Jinshan had PRTs exceeding 15 min, with Xinhai Station in western Chongming reaching the highest at 19.6 min (Fig 9b).

By 18:00, these averages had increased to 12.9 min, 11.4 min, and 13.5 min, respectively. The number of EMS stations with PRTs over 15 min increased to 30 (19.5%), with only two located in the central districts—Shier Station in Huangpu. Among these, 7 EMS stations had PRTs exceeding 18 min: six in Chongming and one in Qingpu. The station with the highest PRT was Changxing Station in Chongming, reaching 26.1 min.

### Multiperiod 12 min and 15 min EAI overlap frequency of all EMS stations.

**Fig 10:**
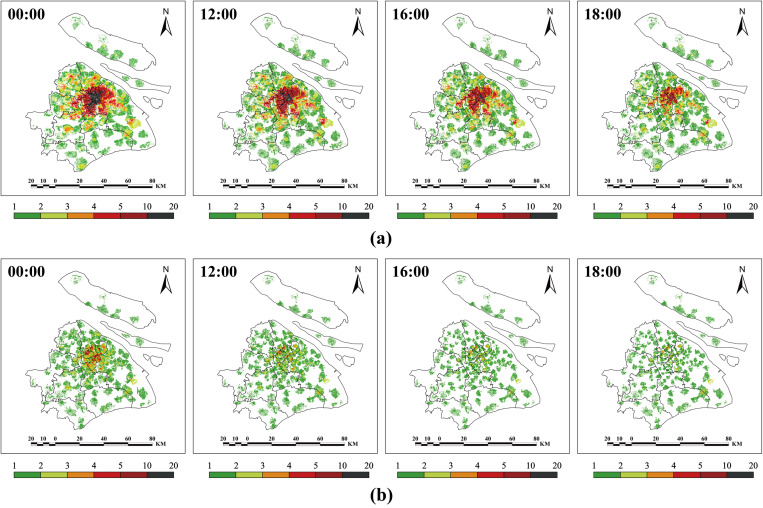
The 12 min, 15 min EAI overlap frequency. (a)The 15 min EAI overlap frequency (b)The 12 min EAI overlap frequency.

This study demonstrated that at 00:00, Shanghai exhibited a significant overlap frequency in the 15 min EAI (Fig 10a). Huangpu District experienced extreme overlap across almost the entire district (10 < frequency ≤ 20), with only the eastern edge showing heavy overlap (5 < frequency ≤ 10). Similarly, the eastern part of Putuo, Jing’an, southern Hongkou, and northern Xuhui also showed extreme overlap in the 15 min EAI, while the remaining areas had heavy overlap. By contrast, Chongming, Jinshan, and Fengxian districts had minimal overlap (frequency ≤ 2) in only a few areas. Shanghai’s 12 min EAI also exhibited significant overlap (Fig 10a). The eastern and northern parts of Huangpu; northern and southern Jing’an; central Putuo; northern Xuhui; and central Yangpu all had heavy overlap in the 12 min EAI.

By 18:00, the overlap frequency of both the 12 min and 15 min EAI decreased to varying extents (Fig 10b). However, the previously mentioned areas with extreme overlap in the 15 min EAI continued to show heavy overlap. Additionally, central Putuo; central Yangpu; northern and southern Jing’an; and eastern Huangpu still exhibited moderate overlap (2 < frequency ≤ 5) in the 12 min EAI.

## Discussion

This study developed an EMS accessibility model by integrating open mapping APIs with the EBK interpolation method. The results indicate that, compared to traditional accessibility calculation methods, this model has three main advantages: (1) Sensitive capability for recognizing multi-period traffic: Compared to traditional accessibility methods, where network analysis relies on standard road speeds and the 2SFCA method utilizes spatial decay functions to estimate driving time costs, this model provides a more precise calculation of multi-period driving times by using web mapping APIs that incorporate real-time speeds across multidimensional road networks. (2) High spatial resolution: We used the EBK interpolation method to spatially predict point-based driving data, significantly improving the spatial resolution of accessibility distribution results. (3) High spatial scalability: The model demonstrates enhanced spatial scalability and innovatively incorporates population distribution data, enabling the accurate calculation of EMS accessibility indicators such as per capita response time (PRT) and EMS accessibility isochrone (EAI) population coverage rates. The former is essential for evaluating citywide EMS efficiency, while the latter is crucial for assessing EMS coverage quality.

The results indicated that EMS accessibility in Shanghai exhibited clear spatiotemporal variation, driven by disparities across urban regions and multi-period traffic conditions. The EMS accessibility in central districts is significantly better than in suburban districts. Some regions of Shanghai, including areas in western and eastern Chongming, eastern Jinshan, northern Jiading, and eastern Pudong, showed significantly lower EMS accessibility. In contrast, the central districts had lower PRT and higher 9–15-min EAI population coverage rate. Furthermore, traffic congestion significantly reduced EMS accessibility across the city. The goal of achieving a PRT of less than 12 minutes was only met during periods of smooth traffic flow, emphasizing the need for further expansion of EMS infrastructure.

The study also revealed significant inequity in EMS accessibility across Shanghai’s urban districts. Thile index results indicated that the large gap between central and suburban districts was the primary source of inequity in Shanghai’s EMS accessibility. Although traffic congestion reduced the disparities between districts, the disparities remained significant. Thus, Shanghai faces substantial equity challenges, particularly in the inadequate EMS accessibility in suburban districts, where populations continue to experience a serious EMS care crisis.

The EMS partitioning analysis, based on the MCR model, showed that the shortest-time service ranges of EMS stations were dynamic due to multiperiod traffic conditions. The study also found that the EMS stations layout in Shanghai was suboptimal. Suburban districts had too few stations, leading to higher PRT and increased resource demand, while central urban districts had an excessively high density, causing frequent overlaps in the 12–15-min EAI. These findings suggested that the EMS station layout had not adequately accounted for population demand or efficiency equity, resulting in a severe imbalance between central and suburban districts.

The study emphasizes that improving EMS accessibility and spatial equity in Shanghai requires the development of a more rational EMS station layout. This can be achieved through two key strategies: (1) Optimizing the existing EMS station layout: To enhance resource utilization efficiency, the 12-min PRT could be used as a benchmark. We could increase the spacing between EMS stations in central districts, particularly in areas such as eastern Huangpu, eastern Putuo, and northern and southern Jing’an, and redistribute resources based on the population covered by the newly defined partitioning areas. Through this, the 12 min EAI population coverage rate can be improved without increasing resources. (2) Expanding EMS infrastructure: New stations should be built in areas with inadequate accessibility, particularly in regions such as western and eastern Chongming, eastern Jinshan, and other previously mentioned areas. By implementing these strategies, a more rational EMS station layout can be established, improving accessibility, reducing spatial inequity, and optimizing resource utilization in Shanghai.

## Conclusions

The results indicated that overall EMS accessibility in Shanghai was highly influenced by traffic conditions, with the PRT being within 12 minutes only during smooth traffic period. Additionally, the current EMS accessibility in Shanghai’s districts exhibits significant inequality. The inequality in PRT and the basic quality rate is primarily between central and suburban districts, while the inequality in the optimal quality rate is evident across all districts. The layout of EMS stations also exhibited irrationality, resulting in EMS stations in suburban districts having higher PRTs, larger service areas, and greater resource demands. Additionally, significant overlaps in the 12 min and 15 min EAI were observed in central districts, pointing to potential resource wastage, while suburban districts, particularly Chongming, Jinshan, and Fengxian, showed lower population coverage rates and inadequate EMS resources. These findings highlight the urgent need to optimize EMS station allocation, prioritize suburban areas, and address inefficiencies in the current EMS layout to ensure equitable access to emergency services for all residents.

Furthermore, this method has significant potential in the field of accessibility research and can be extended to other cities. It is also applicable to other urban public services, such as hospitals and fire stations. This broad applicability underscores the versatility and impact of the approach developed in this study.

## Limitations

The EMS driving-time data were sampled on a single working day, potentially overlooking long-term EMS conditions. Nevertheless, since LandScan provides average population distribution over the year, it cannot capture real-time population demand. This limitation introduces uncertainty, as our study can only estimate the overall probability of multi-period EMS demand, potentially leading to inaccuracies in the results. Future research could involve continuous data sampling and integration of real-time population analysis using mobile signal data to enhance study accuracy.
